# Diet variation and trophic impact of weakfish, *Cynoscion regalis*, within multiple marine habitats of the eastern United States

**DOI:** 10.1111/jfb.15897

**Published:** 2024-08-12

**Authors:** Angel Reyes Delgado, Brian E. Smith

**Affiliations:** ^1^ Department of Natural and Agricultural Sciences University of Maryland Eastern Shore Princess Anne Maryland USA; ^2^ National Marine Fisheries Service Northeast Fisheries Science Center Woods Hole Massachusetts USA

**Keywords:** continental shelf, ecosystem, Northwest Atlantic, piscivore, predation, trophic ecology

## Abstract

Weakfish (*Cynoscion regalis*) is not federally managed but feeds on species of management and ecological interest. We examined the trophic ecology of weakfish in Chesapeake Bay and the coastal and offshore waters of the eastern United States. For these areas, we determined the dominant prey of weakfish; identified how much diet variation was explained by the factors: season, size class, and year; and quantified prey biomass removed by weakfish from 2007 to 2019. In general, diet composition was mostly dominated by Engraulidae, Osteichthyes (bony fishes), and Mysidacea, and significantly varied by season and size class in Chesapeake Bay and coastal waters, although this was less dramatic in Chesapeake Bay. The total amount of variance explained by the three factors was 23.1% (Chesapeake Bay) and 14.7% (coastal waters), with year not being a significant factor in explaining weakfish diet variation for these areas. Weakfish total prey biomass removal occurred primarily in coastal waters in the fall and small size class (annual mean: approximately 41,038 t; maximum: approximately 63,793 t). Highly opportunistic feeders, weakfish cannibalism also played an essential part of their diet. These results have implications for fisheries and ecosystem management of weakfish when considering ecological interactions in regulatory approaches, such as recruitment and cannibalism, competition with federally managed fishes, and the natural mortality of their prey.

## 1 INTRODUCTION

*Weakfish Cynoscion regalis* (Bloch and Schneider 1801) is a *Sciaenid* distributed along the eastern, southern, and western coastal United States (Bailey et al., 1970). In the Northwest Atlantic, it inhabits regions ranging from Nova Scotia, Canada, southward to Florida (Wilk, 1978), but more commonly from New York to North Carolina (Hildebrand & Schroeder, 1928). During migration, weakfish enter shallow regions within estuaries along inner‐continental shelf habitats for feeding, spawning, and rearing juveniles (Turnure et al., 2015) in the spring, then return to deeper and offshore waters in the fall (Mercer, 1989; Shepherd & Grimes, 1984). Foraging throughout the water column, weakfish are known mainly as opportunistic predators with mixed prey preference (Parthree, 2005; Willis et al., 2015) that rely more on fish and demersal prey as they grow older (Hartman & Brandt, 1995). Their diverse diet is composed of several species of bony fish (including cannibalism) and small crustaceans. It includes prey of commercial interest at both regional and federal levels (Garrison & Link, 2000; Hartman & Brandt, 1995; Nemerson & Able, 2004; Parthree, 2005; Smith & Link, 2010; Taylor, 1987; Wilk, 1978; Willis et al., 2015; Wuenschel et al., 2013) and varies with ontogeny, season, and prey availability (Hartman & Brandt, 1995b; Stehlik et al., 2017). As juveniles, weakfish have been seen to selectively prey on smaller items (e.g., Mysidacea, sevenspine bay shrimp *Crangon septemspinosa* [Say 1818]; Lankford & Targett, 1997) while older individuals shift to piscivory, particularly larger fishes (e.g., Atlantic menhaden *Brevoortia tyrannus* [Latrobe 1802], spot *Leiostomus xanthurus* [Lacepède 1802]; Hartman & Brandt, 1995). Owing to their predatory nature, weakfish are in competition, especially in the Chesapeake Bay and possibly within nearby estuaries, with other piscivores, such as striped bass *Morone saxatilis* (Walbaum 1792), bluefish *Pomatomus saltatrix* (Linnaeus 1766), summer flounder *Paralichthys dentatus* (Linnaeus 1766), and larger individuals of their same species (Hartman, 1993; Hartman & Brandt, 1995; Latour et al., 2008; Wilk, 1978; Wuenschel et al., 2013). As a highly piscivorous‐shrimp crustacivore and top predator in systems such as the Chesapeake Bay (Hartman & Brandt, 1995), weakfish play a crucial role in the nearshore food web (Willis et al., 2015). They have the potential to regulate energy flow (top‐down control) or to be influenced by changes in production (bottom‐up control) at lower trophic levels (Carpenter et al., 1985, 1987).

The weakfish stock is managed by the Atlantic States Marine Fisheries Commission (ASMFC) under Amendment 4 of the Weakfish Fishery Management Plan (ASMFC, 2021; Brust et al., 2016). Because establishing stock boundaries is challenging due to few genetic studies (Cordes & Graves, 2003; Graves et al., 1992), and limited tagging and meristic/life‐history (Crawford et al., 1988) data available, weakfish are assessed and managed as a single stock across their known range (ASMFC, 2016). Current stock biomass remains low and has not recovered from declines in the late 1990s, reaching an all‐time low in 2003 (ASMFC, 2019). Although weakfish stock status remains depleted, the most recent assessment suggests overfishing is not occurring (ASMFC, 2021). It is believed that natural mortality (starvation, predation, and disease) in the mid‐1990s, together with fishing mortality, even though harvest levels have been low in recent years, largely caused weakfish biomass and size structure to decline greatly and to remain low (ASMFC, 2019). Determining the natural mortality of commercial fish stocks from predation is an ongoing challenge even where extensive time series of empirical feeding data exists (Deroba, 2018; Mannini et al., 2020); still, quantifying predatory removals, including cannibalism, has been useful for understanding fish population dynamics (e.g., recruitment; Jurado‐Molina et al., 2006; Link et al., 2012).

Weakfish are regulated at a regional level but are mainly piscivorous and feed on species that are regulated at a federal level and of commercial interest. Previous work on weakfish diet variability among these essential prey and across multiple marine environments is limited for the Northwest Atlantic. Most of these studies have identified commercially important prey in weakfish, including cannibalism (e.g., Atlantic menhaden, butterfish *Peprilus triacanthus* [Peck 1804], scup *Stenotomus chrysops* [Linnaeus 1766], Atlantic herring *Clupea harengus* [L.], Silver hake *Merluccius bilinearis* [Mitchill 1814], spot, and *Loligo* spp.[Lamarck 1798]) for multiple areas scattered across the eastern U.S. coast (e.g., Garrison & Link, 2000; Hartman & Brandt, 1995; Parthree, 2005; Smith & Link, 2010; Taylor, 1987; Willis et al., 2015; Wuenschel et al., 2013). However, they did not examine their diet over large spatial and temporal scales. Addressing this gap will provide information for stock assessments about the predatory impact of weakfish on several of these prey that are managed at federal and regional levels and for understanding the role of weakfish cannibalism on recruitment to quantify weakfish natural mortality. Therefore, our objectives were to (1) determine the dominant prey in weakfish diet for three areas of the eastern United States, (2) identify whether season, size class, and year factors explain variations in the percentage of diet composition for each area, and (3) quantify how much prey biomass is removed by weakfish by season, size class, and year for each area.

## 2 METHODS

### 2.1 Ethics statement

The sampling of specimens in this study complied with US Animal Welfare Act laws, guidelines, and polices as approved by the National Oceanic and Atmospheric Administration (NOAA) Fisheries, the Atlantic States Marine Fisheries Commission (ASMFC), and the Commonwealth of Virginia, which determined that no animal use protocol was required.

### 2.2 Data

**FIGURE 1:**
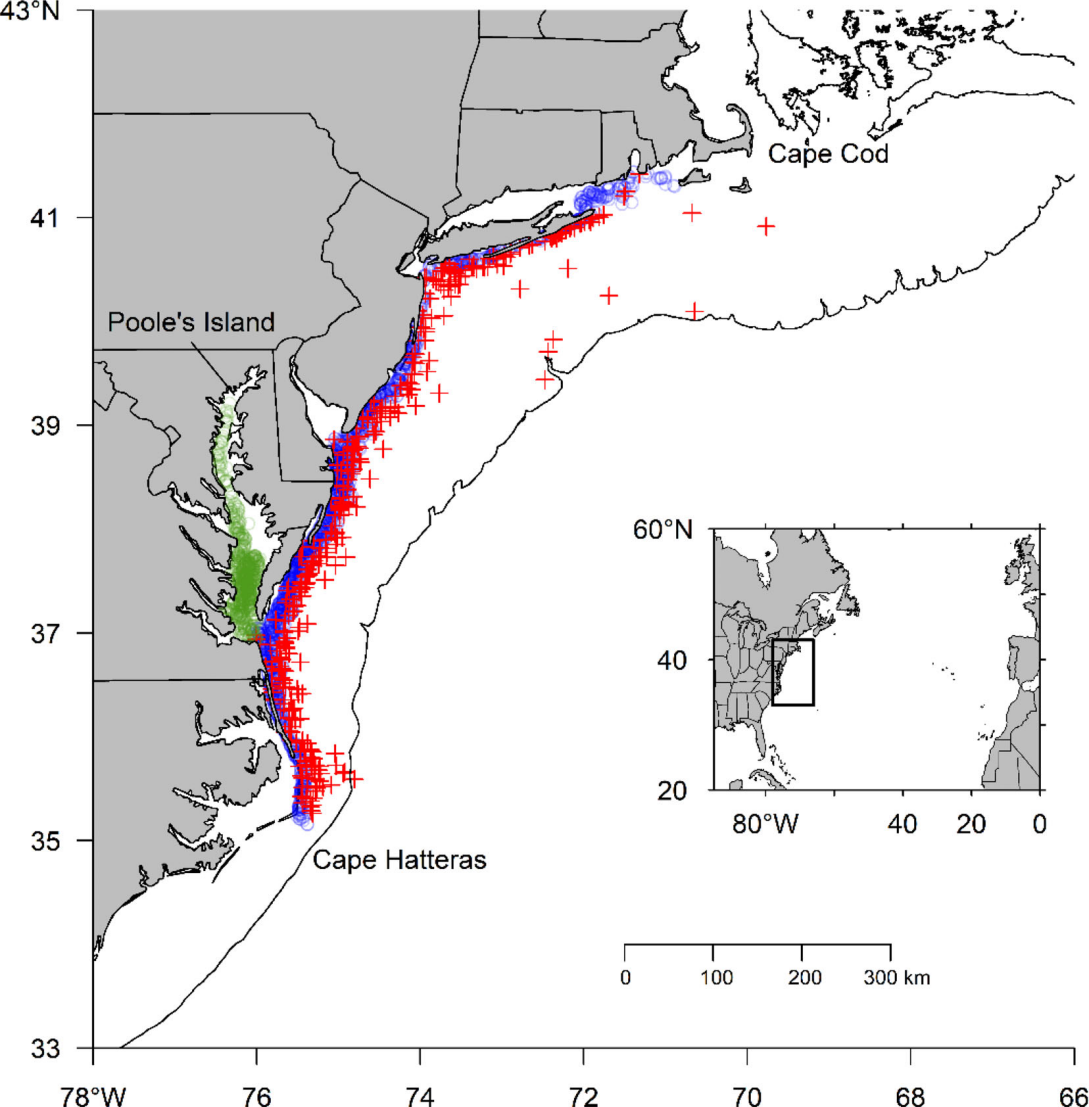
Map of weakfish diet sampling stations by CHESMMAP, NEAMAP, and National Marine Fisheries Service (NMFS) surveys between Cape Hatteras and Cape Cod of the Northeast United States. Green circles = CB (CHESMMAP); blue circles = inshore (NEAMAP); red “+” = offshore (NMFS). Bathymetry shown is 200 m.

We used diet data from three monitoring programmes of the eastern United States that conduct bottom trawl surveys to monitor fish communities following a depth‐stratified random sampling design. These programmes included (1) Chesapeake Bay Multispecies Monitoring and Assessment Program (CHESMMAP; 2007–2018), (2) Northeast Area Monitoring and Assessment Programme (NEAMAP; 2007–2019), and (3) National Marine Fisheries Service (NMFS; 2007–2019) bottom trawl survey (Figure 1). CHESMMAP performs bottom trawls in Chesapeake Bay to sample around 12 different juvenile and adult fish species from Poole's Island, Maryland (north) to the Bay's mouth (south) at depths ranging between 3 m to more than 15 m in the spring (March and May), summer (July), and fall (September and November; Bonzek et al., 2022). With the area divided into five regions of around 30 latitudinal minutes of difference between them, the region has 80 sample sites. NEAMAP is a bottom trawl survey that targets late‐juvenile and adult stages of approximately 28 finfish species (Latour et al., 2021). Fish stomach samples were collected in spring (April–May) and fall (October–November) from 150 sites distributed along the coast of Cape Cod, Massachusetts, to Cape Hatteras, North Carolina, at depths ranging between 6 and 30 m. NMFS uses a bottom trawl survey to collect fish and sample stomachs from 60 species of juvenile and adult fishes at approximately 350–400 stations with depths ranging between 8 and 400 m from Nova Scotia to Cape Hatteras, North Carolina, in winter (February), spring (March–May), and fall (September–November; 2007–2019; Politis et al., 2014; Smith & Link, 2010). Weakfish were collected, and their stomach contents were identified to the lowest taxonomic level possible using microscopic (CHESMMAP and NEAMAP; Bonzek et al., 2007, 2022) or macroscopic (NMFS; Smith & Link, 2010) examination. Prey mass (CHESMMAP and NEAMAP) or volume converted to mass (1:1.1 [volume‐to‐mass]; NMFS) was applied based on linear regression (*r* ^2^ = 0.906; *p* < 0.0001) by Link and Almeida (2000) to quantify prey taxa biomass throughout the time series for each programme.

### 2.3 Diet analysis

The adequacy of diet sampling for each area was assessed with trophic diversity (Shannon H) curves (Alonso‐Koen et al., 2002; Ferry & Caillet, 1996; Smith et al., 2013), considering three factors: (1) season (spring and fall), (2) size category based on Lmax (small [1–25 cm] and medium [26–50 cm]; large ones were excluded because of data deficiency), and (3) years 2007–2019. The order of stomachs was randomized 100 times, and asymptotic diversity was reached when the difference between the final diversity value and the average of the five preceding values was less than 0.05, which was similar to Alonso‐Koen et al. (2002) but slightly less conservative than Smith et al. (2013).

To examine important weakfish prey, we estimated diet composition by mass and frequency of occurrence using a two‐stage cluster sampling estimator (Buckel et al., 1999; Latour et al., 2008; Link & Almeida, 2000). Due to the survey design of each monitoring platform, sampling weakfish with bottom trawls produces clusters of samples that are not equally representative of weakfish feeding. To account for this, the diet data were weighted by the number of weakfish caught per tow rather than a simple average across all weakfish stomach samples. The resulting average biomass of prey taxa estimates what an individual weakfish eats. Barplots were created to determine the top prey in each area as a percentage of the total sum of biomass or percentage of frequency of occurrence per prey taxa. Rare prey that constituted less than 1% by mass were excluded. Top prey constituted ~90% of total prey by mass for each area. Prey taxa abbreviations and definitions are presented in Table 1.

**TABLE 1:** Abbreviations and scientific names of prey items classified by category.

Abbreviation	Prey item	Category
Acetes	*Acetes* spp. (H. Milne‐Edwards 1830)	Shrimps
Alosa	*Alosa* spp. (Linck 1790)	Fish
Ammody	*Ammodytes* spp. (L.)	Fish
Animal	Animal remains	Other
Animal.1	Animal tubes	Other
Ascidi	Ascidiacea	Other
Bairdi	*Bairdiella chrysoura* (silver perch) (Lacépède 1802)	Fish
Bivalv	Bivalvia	Bivalves
Brevoo	*Brevoortia tyrannus* (Atlantic menhaden)* (Latrobe 1802)	Fish
Centro	*Black sea bass Centropristis striata* * (L.)	Fish
Cephal	Cephalopoda	Mollusks
Clupe	Clupeidae	Fish
Cnidar	Cnidaria	Other
Copepo	Copepoda	Crustacean
Crango	Crangonidae	Shrimps
Crusta	Crustacea	Crustacean
Crusta.1	Crustacean shrimp	Shrimps
Cynosc	Weakfish Cynoscion regalis* (Bloch and Schneider 1801)	Fish
Decapo	Decapoda	Crustacean
Decapo.1	Decapoda crab	Crustacean
Decapo.2	Decapoda larvae	Crustacean
Engrau	Engraulidae (anchovies)	Fish
Ensis	*Ensis directus* (Conrad 1843)	Bivalves
Etropu	*Etropus microstomus* (Gill 1864)	Fish
Etrume	*Etrumeus teres* (DeKay 1842)	Fish
Euphau	Euphausiacea	Shrimps
Fish.l	Fish larvae	Fish
Gammar	Gammaridea	Amphipoda
Gastro	Gastropoda	Mollusks
Leiost	Spot Leiostomus xanthurus* (Lacepède 1802)	Fish
Limulu	*Limulus polyphemus* (L.)	Crustacean
Loligo	*Loligo* spp.* (Lamarck 1798)	Squids
Menidi	*Menidia* spp. (Bonaparte 1836)	Fish
Mentic	*Menticirrhus* spp. (Gill 1861)	Fish
Merluc	Silver hake Merluccius bilinearis* (Mitchill 1814)	Fish
Microp	Atlantic croaker Micropogonias undulatus* (Linnaeus 1766)	Fish
Miscell	Miscellaneous	Other
Mollus	Mollusca	Mollusks
Mollus.1	Mollusca shell	Mollusks
Mysida	Mysidacea	Shrimps
Osteic	Osteichthyes (bony fish)	Fish
Pandal	Pandalidae	Shrimps
Penaei	Penaeidae	Shrimps
Pepril	Butterfish Peprilus triacanthus* (Peck 1804)	Fish
Planta	Plantae	Other
Polych	Polychaeta	Worms
Pomato	Bluefish Pomatomus saltatrix* (Linnaeus 1766)	Fish
Priono	*Prionotus* spp. (Lacepède 1801)	Fish
Sand	Sand	Other
Sciaen	Sciaenidae	Fish
Scombe	Atlantic mackerel Scomber scombrus* (L.)	Fish
Selene	*Selene setapinnis* (Mitchill 1815)	Fish
Soleno	Solenoidea	Bivalves
Stenot	Scup Stenotomus chrysops* (Linnaeus 1766)	Fish
Stomat	Stomatopoda	Shrimps
Urophy	*Urophycis* spp. (Gill 1863)	Fish

A canonical correspondence analysis (CCA) was performed to examine how much diet variation was explained for weakfish in each area by the factors: season, size category, and year. CCA allows ecological interpretations of species assemblages due to its feature that orders species along the canonical axes following their ecological optima (Borcard et al., 2011). The explanatory factors were expanded to “dummy” variables reflecting size categories (small and medium), season (spring and fall), and years (2007–2019). Explanatory factors for the offshore area excluded season given that weakfish were primarily observed in the fall. The response variables were the weighted mean percentages of prey by mass and log transformed (ln[*x* + 1]) to account for any non‐normality in the distribution of prey percentages. Similar standards to objective one were considered to exclude rare prey prior to analyses. This analysis was conducted using the Vegan R package cca function, and we tested whether these factors would explain significant amounts of diet variance relative to random chance with the Vegan R package anova.cca function (version 4.2.1; R Core Team, 2022). Biplots were used to examine dietary trends with the three explanatory factors within each area. Prey taxa abbreviations and definitions are presented in Table 1. Although arguably not conservative, we chose to retain the existing prey categories rather than “lump” them into fewer taxa, as this would limit statistical detection of diet variability.

To quantify the trophic impact of weakfish over time for each area, we included the same factors (seasons, size categories, and years) that resulted in adequate numbers of weakfish stomachs available based on the previous trophic diversity curves. The gastric evacuation rate method (Eggers, 1977; Elliot & Persson, 1978) was used to calculate per capita consumption by weakfish for each area. Per capita consumption rate (*C*_*p*,*t*_) was calculated on a daily basis:

(1) $$C_{p,t,s}=24*E_{p,t,s}*D_{p,t,s}$$

where *p* is each prey, *t* is the season, and *s* is the size category. The 24 represents the number of hours in a day. *D*_*p*,*t*,*s*_ represents the seasonal mean amount of prey (g) consumed by an individual weakfish of size category *s* using the same two‐stage cluster sampling estimator used previously (Buckel et al., 1999; Latour et al., 2008; Link & Almeida, 2000), assuming a continuous feeding rate for 24 h. *E*_*p*,*t*,*s*_ represents the evacuation rate, calculated as follows:

(2) $$E_{p,t,s}=\alpha e^{{\mathrm{\beta T}}_{p,t,s}}$$

where parameters *α* (intercept) and *β* (slope) were set as 0.004 and 0.115, respectively, similar to other studies (Overholtz et al., 1999, 2000; Tsou & Collie, 2001a, 2001b). *T*_*p*,*t*,*s*_ is defined as the mean ambient temperature weighted by the stratum area of occurrence of weakfish from their respective trawls for each season, size category, year, and monitoring programme (Bonzek et al., 2007; Bonzek et al., 2022; Taylor & Bascuñán, 2000). The daily consumption rate of each prey by season was scaled up to a quarter‐year estimate by multiplying with 91.25 (days in a quarter year). Estimates were left as seasonal rates (not summed as annual estimates) to show within‐year temporal variability in feeding and data availability.

Seasonal population‐level consumption for each size category was estimated by scaling the seasonal per capita consumption rate by the seasonal population abundance of weakfish (*P*_*t*,*s*_), similar to Link and Sosebee (2008) and Rowe and Smith (2022). *P*_*t*,*s*_ was calculated by scaling the stratified mean weakfish abundance per tow by the area swept for weakfish across the mainland to offshore gradient for each area, season, size category, and year. For the vessel and gear changes that occurred over the time period with the NMFS survey, calibration coefficients for weakfish were provided by Miller et al. (2010). Area swept per tow for each survey was assumed to be 0.016 km^2^ (CHESMMAP), 0.022 km^2^ (NEAMAP), or 0.0384 km^2^ (NMFS) based on net mensuration gear. Catchability of weakfish was assumed to equal 1.0 for all surveys.

Seasonal consumption rates in tonnes per year and size category for the weakfish population were calculated by multiplying the seasonal population abundance with the seasonal per capita consumption rate. Uncertainty associated with trophic impact analyses was reported with 95% CIs based on 1000 random observations from gamma distributions for each input parameter of Equations (1) and (2) (*α*, *β*, *T*_*p*,*t*,s_, and *D*_*p*,*t*,*s*_) and population abundance (*P*_*t*,*s*_).

## 3 RESULTS

**FIGURE 2:**
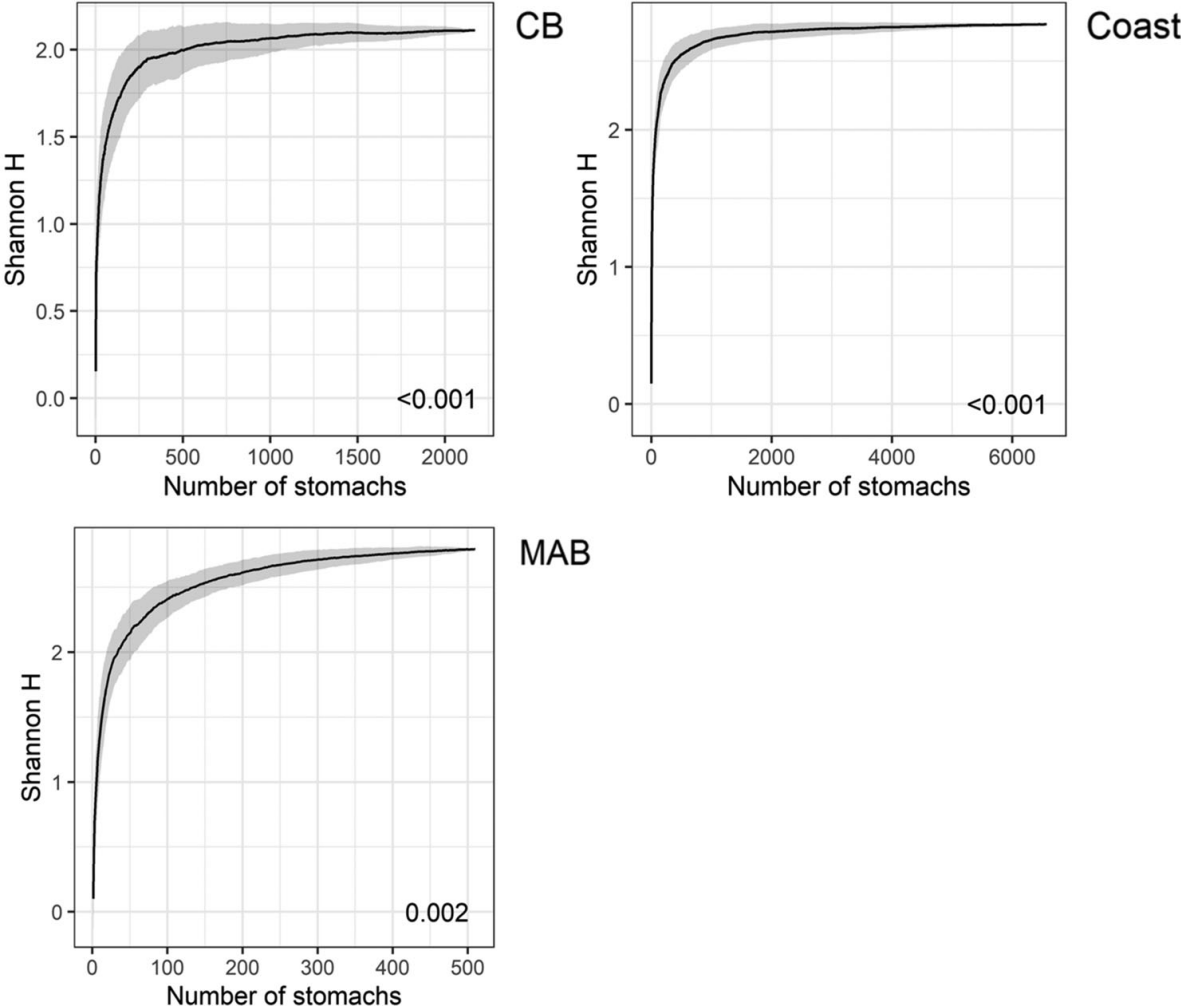
Trophic diversity curves for a number of stomachs of weakfish by area. Black line represents mean Shannon H. Gray shading denotes standard deviation. Labeled values indicate asymptotic diversity as the difference between the last value and average of the five preceding values. CB, Chesapeake Bay; coast, inshore; MAB, Mid‐Atlantic Bight/offshore.

Trophic diversity curves for each of the three areas (CB, inshore, and offshore) and by the factor combinations for each area by season, size category, and year reached asymptotes. This indicated that adequate numbers of stomachs were available to examine weakfish diet compositions and diet variation among these factors by area (510–6570) (Figure 2) and by the factor combinations (12–403) (Figures [Link], [Link]).

**FIGURE 3:**
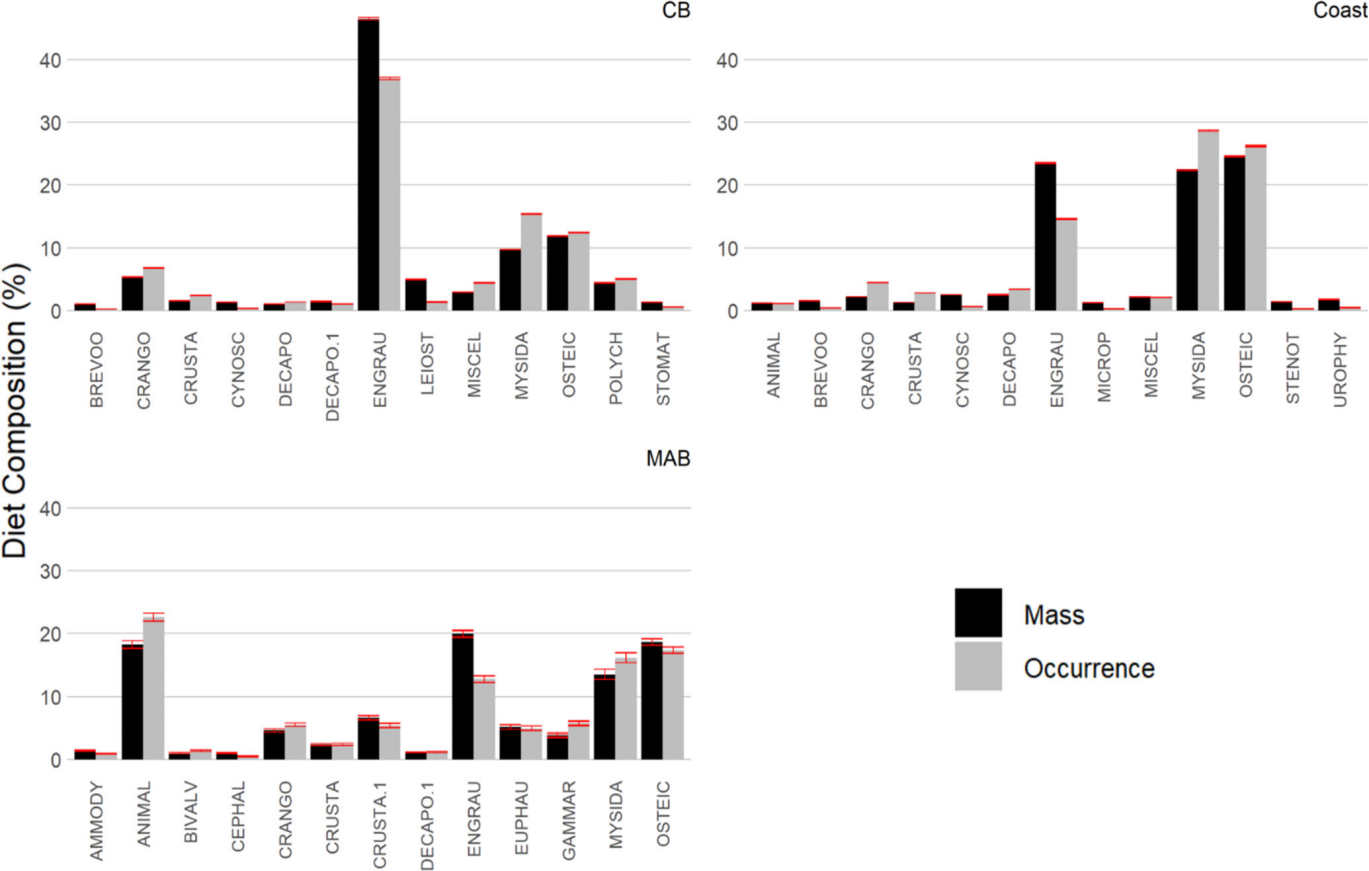
Barplots of diet composition (mass and frequency of occurrence) for major prey types of weakfish by area. Red error bars denote 95% confidence intervals. Prey taxa abbreviations and definitions are presented in Table 1. CB, Chesapeake Bay; coast, inshore; MAB, Mid‐Atlantic Bight/offshore.

Overall, Engraulidae, Osteichthyes (bony fish), and Mysidacea were the most dominant identifiable prey for each area by percentage mass and percentage frequency of occurrence (Figure 3). In CB, Engraulidae was the dominant prey, comprising approximately four times as much mass and twice as much occurrences as Mysidacea and bony fish. Inshore and offshore, weakfish mainly fed on these three primary prey in relatively similar proportions, with the exception of higher unidentifiable animal remains observed offshore.

### 3.1 Diet variation

**FIGURE 4:**
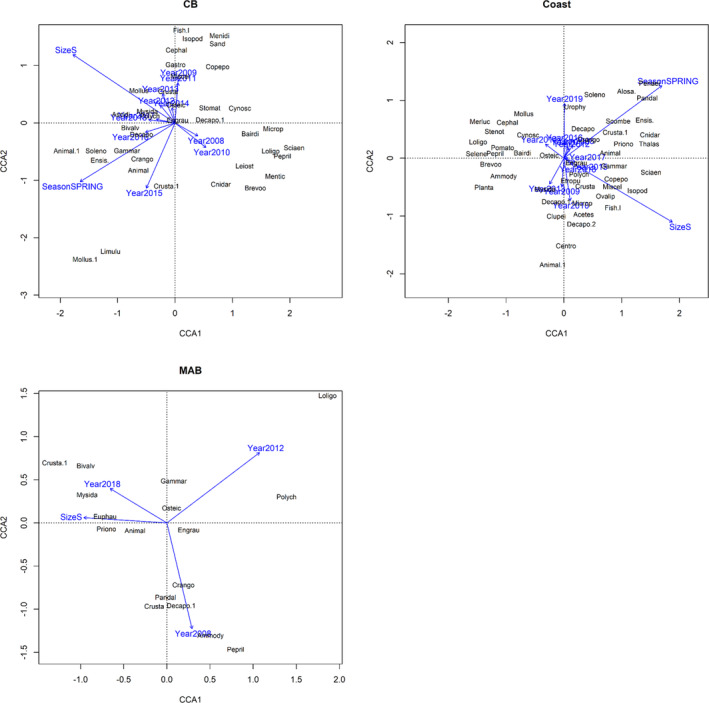
Canonical correspondence analysis (CCA) biplots of the major prey types of weakfish by area. Prey taxa abbreviations and definitions are presented in Table 1. CB, Chesapeake Bay; coast, inshore; MAB, Mid‐Atlantic Bight/offshore. Centroids for the explanatory factors and corresponding elements are shown in blue. S = small.

The CCA analysis by area showed marginally significant (CB: 999 permutations, DF = 13, 13, *F =* 1.2, *p* = 0.07), significant (inshore: 999 permutations, DF = 14, 26, *F =* 1.4, *p =* 0.001), or no significant results (offshore: 719 permutations, DF = 4, 1, *F =* 1.2, *p =* 0.27) under the full models across the explanatory factors: size category, season, and year. Similarly, size category (CB: DF = 1, 13, *F =* 2.1, *p =* 0.004; inshore: DF = 1, 26, *F* = 2.8, *p* = 0.001) and season (CB: DF = 1, 13, *F =* 2.7, *p =* 0.001; inshore: 1, 26, *F* = 3.2, *p* = 0.001) were significant factors in explaining the diet variation in weakfish for CB and inshore waters. Biplots for the three areas are shown in Figure 4. Year (CB: DF = 11, 13, *F =* 0.98, *p =* 0.56; inshore: DF = 12, 26, *F* = 1.1, *p* = 0.13) was not a significant factor. For CB and inshore waters, season and size category explained 23.1% (CB) and 14.7% (inshore) of the diet variability observed, respectively. The predation of fish prey, such as Atlantic menhaden, spot, *Menticirrhus* spp. (Gill 1861), and butterfish, was associated with medium‐sized weakfish in CB in contrast to smaller custacean prey (Mysidacea), Mollusca, fish larvae, and smaller fishes (e.g., *Menidia* spp. [Bonaparte 1836]) in the diets of small‐sized weakfish. Similarly, medium‐sized weakfish diets in inshore waters included fish prey—Atlantic menhaden, silver hake, scup, *Loligo* spp.—and were more associated with cannibalism (weakfish), whereas small‐sized weakfish again ate various crustaceans, fish larvae, and also clupeids. Seasonal shifts in diet between spring and fall were also somewhat similar between CB and inshore waters with sevenspine bay shrimp eaten in the spring, Decapoda crabs eaten in the fall, and Engraulidae eaten in both seasons. Spring‐CB diets also included some invertebrate benthos (various bivalves and polychaetes), and the fall had fish larvae and *Menidia* spp. Inshore‐spring diets had Alosine fishes along with invertebrate benthos (various bivalves and crustaceans), and fall diets had more fishes (*Ammodytes* spp. L., scup, and black sea bass *Centropristis striata* [Linnaeus 1758]), as well as Decapoda larvae and Mysidacea.

### 3.2 Trophic impact

**FIGURE 5:**
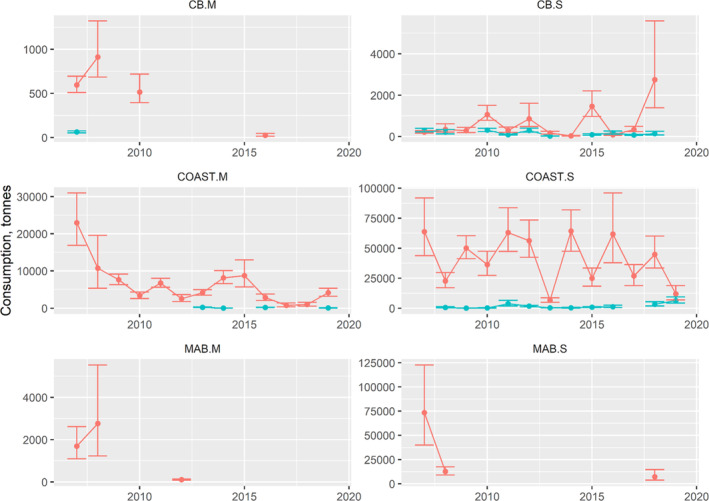
Time series of total prey biomass removed by weakfish in Chesapeake Bay (CB), inshore (COAST), and offshore (MAB). Error bars denote 95% confidence intervals. Colors correspond to fall (red) and spring (blue). M, medium; S, small.

Most of the prey biomass removal occurred inshore and in the fall season by small weakfish (≤25 cm; Figure 5). For all the prey included in the trophic impact analysis, Table 2 lists the corresponding time series mean, minimum, and maximum amounts of each prey, as well as the combined amounts of all prey, by area and size category for the fall season. Total prey biomass showed some decreases early in the time series, but this was only clear for inshore‐medium‐sized weakfish with sufficient data each year (Figure 5). Data for CB‐medium‐sized weakfish and the offshore waters were much more limited but had similar decreases in total prey biomass consumed in 2007–2019. Aside from a recent increase (2018) in fall total prey biomass consumed by CB‐small weakfish, the time series was mostly stable with an average of approximately 657 t of prey per year, and this stability was also seen in spring (averaging 170 t per year). Small weakfish feeding in fall‐inshore waters hovered around 41,000 t per year with the exception of 2013 (6614 t) and 2019 (11,936 t). For these same weakfish, the spring had much less feeding with an average of 1729 t per year.

**TABLE 2:** Minimum (min), mean, and maximum (max) prey biomass values per area for small‐sized weakfish followed by medium‐sized weakfish in parenthesis.

	CB biomass (tonnes)			Coast biomass (tonnes)			MAB biomass (tonnes)		
Prey item	~Min	~Mean	~Max	~Min	~Mean	~Max	~Min	~Mean	~Max
*Ammodytes* spp.				245.4 (53.3)	508.3 (1484.9)	787.8 (6215.5)	1404.2 (2355.1)	1404.2 (2355.1)	1404.2 (2355.1)
*Brevoortia tyrannus* (Atlantic menhaden)*	4.0 (13.8)	59.7 (120.3)	115.4 (237.9)	189.0 (13.0)	1409.1 (1020.4)	3740.6 (4513.0)			
*Centropristis striata* (black sea bass)*				165.1 (11.8)	2477.7 (11.8)	4790.3 (11.8)			
Clupeidae	4.8	4.8	4.8	279.3 (0.4)	3317.6 (157.7)	6355.9 (414.7)			
*Cynoscion regalis* (weakfish)*	17.5 (173.6)	30.3 (173.6)	43.1 (173.6)	20.2 (0.8)	1285.9 (2297.7)	4481.0 (12,517.4)	(324.7)	(324.7)	(324.7)
Engraulidae (anchovies)	51.2 (27.2)	997.1 (72.5)	4694.7 (164.4)	3870.8 (95.7)	32,208.2 (3248.3)	91,415.1 (23,223.0)	161.6 (4.7)	28,390.7 (1320.3)	81,086.4 (2755.5)
*Leiostomus xanthurus* (spot)*	19.8 (571.0)	303.5 (792.0)	587.3 (1071.1)	(40.4)	(103.5)	(190.4)			
*Loligo* spp.*	1.7 (1.1)	1.7 (1.1)	1.7 (1.1)	23.8 (1.9)	83.1 (676.2)	216.5 (2262.5)	(112.5)	(112.5)	(112.5)
*Merluccius bilinearis* (silver hake)*				68.9 (84.0)	323.6 (548.1)	578.2 (1273.1)			
Mysidacea	0.4 (0.3)	26.6 (2.6)	111.2 (4.9)	1502.3 (0.2)	12,160.9 (177.2)	30,733.4 (1197.5)	995.0 (115.9)	6747.3 (115.9)	11,062.7 (115.9)
Other prey (e.g., bony fish, unidentified cephalopods, unidentified crustaceans, and animal remains)	5.5 (15.8)	139.8 (121.3)	377.9 (232.1)	4616.9 (334.0)	27,419.9 (3901.7)	66,299.9 (9112.5)	1875.9 (82.2)	22,062.3 (445.9)	46,746.0 (970.8)
*Peprilus triacanthus* (butterfish)*	(18.9)	(18.9)	(18.9)	79.6 (1.3)	553.9 (330.4)	1480.1 (1443.5)	(327.0)	(327.0)	(327.0)
*Stenotomus chrysops* (scup)*				28.0 (2.1)	854.3 (106.9)	1680.5 (497.6)			
Total prey	32.2 (26.5)	657.3 (512.7)	2748.8 (912.1)	6613.7 (763.5)	41,038.3 (6459.5)	64,340.0 (22,965.5)	7119.4 (110.4)	31,075.5 (1524.5)	73,465.4 (2766.8)

*Note*: Table represents fall, where most of the diet data was available. Prey of commercial interest are highlighted with an asterisk (*). Abbreviations: CB, Chesapeake Bay; MAB, offshore.

### 3.3 Prey‐specific impact

**FIGURE 6:**
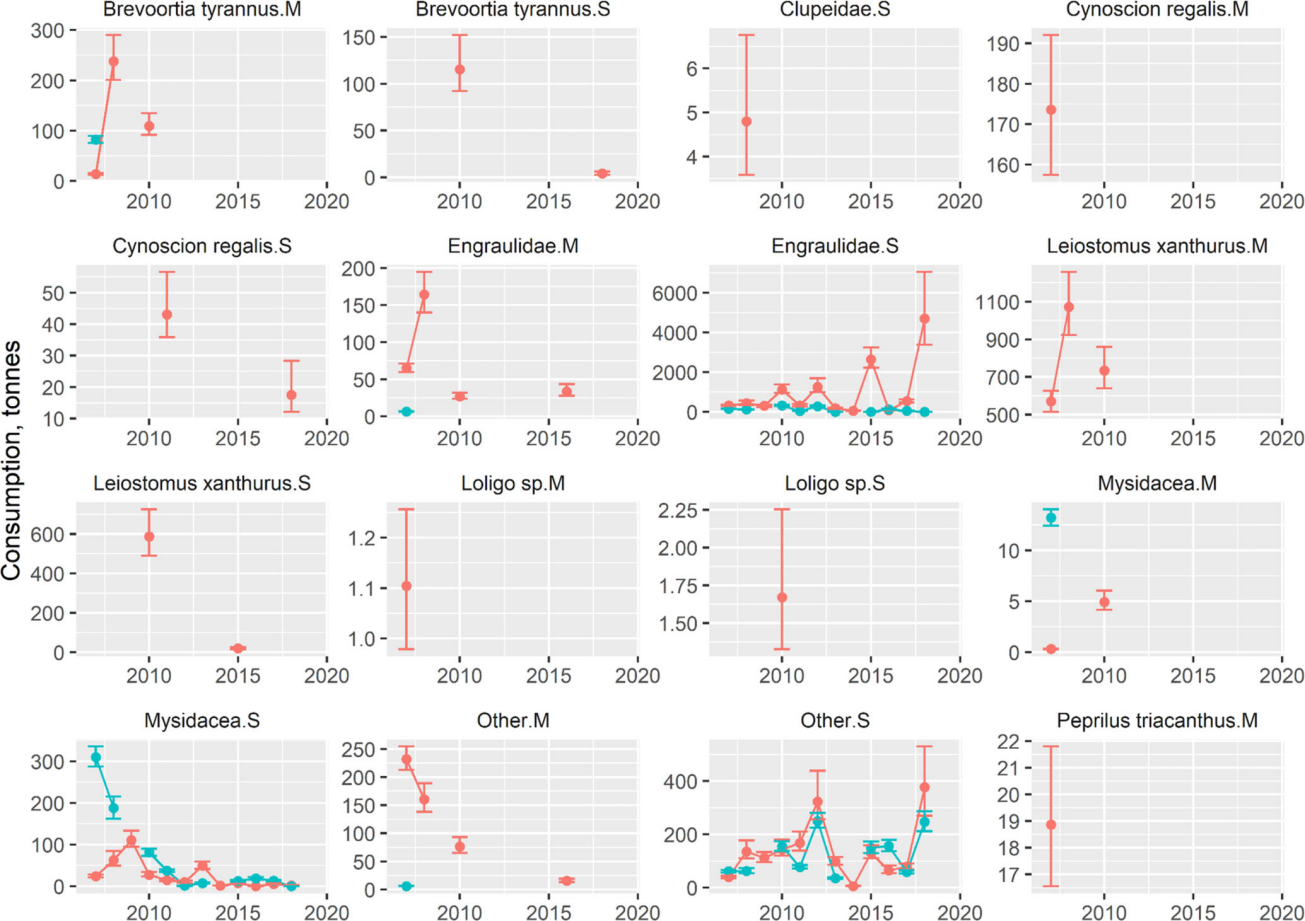
Time series of prey biomass removed by weakfish in the Chesapeake Bay. Error bars denote 95% confidence intervals. Colors correspond to fall (red) and spring (blue). M, medium; S, small.

**FIGURE 7:**
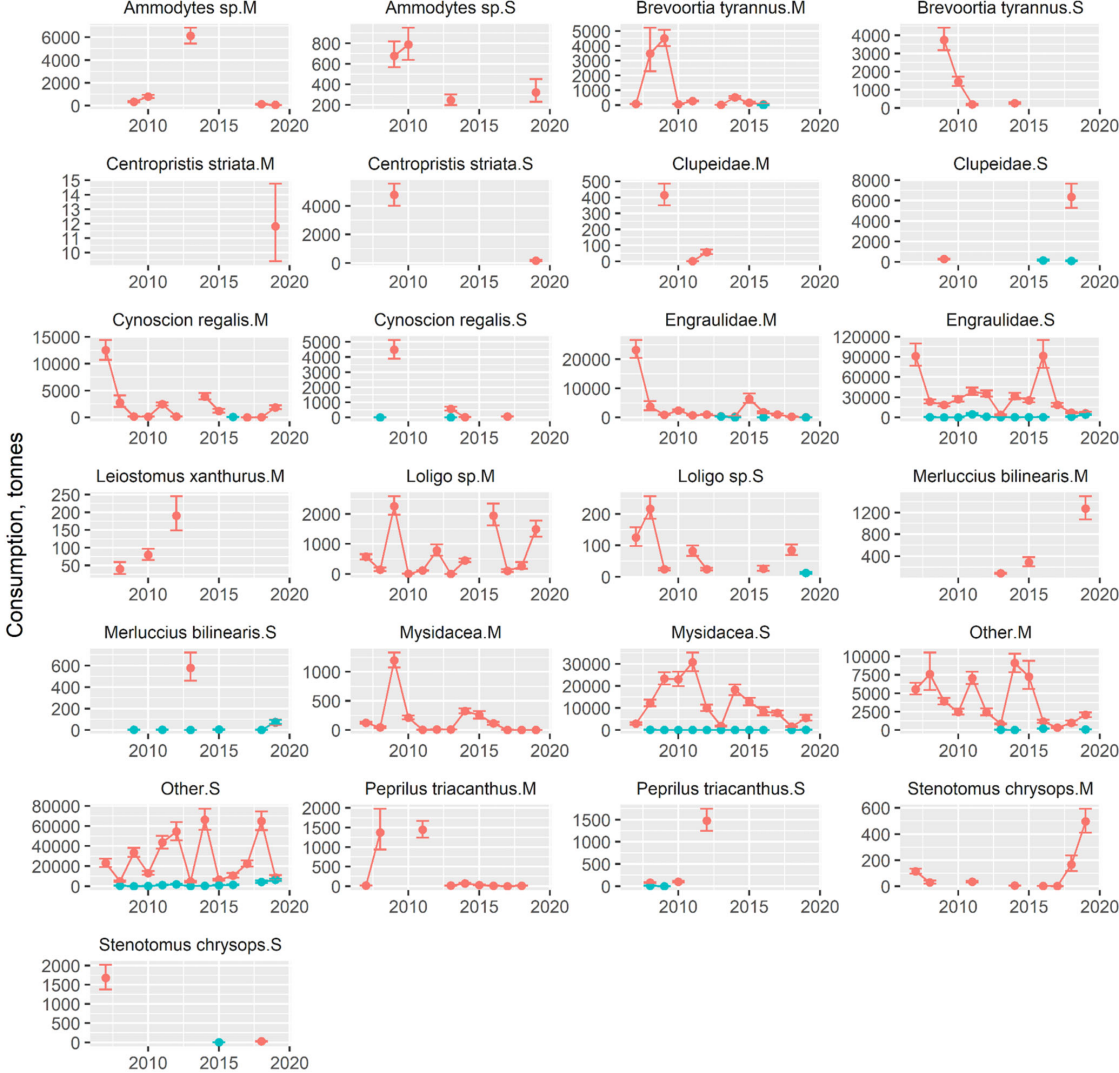
Time series of prey biomass removed by weakfish inshore (COAST). Error bars denote 95% confidence intervals. Colors correspond to fall (red) and spring (blue). M, medium; S, small.

**FIGURE 8:**
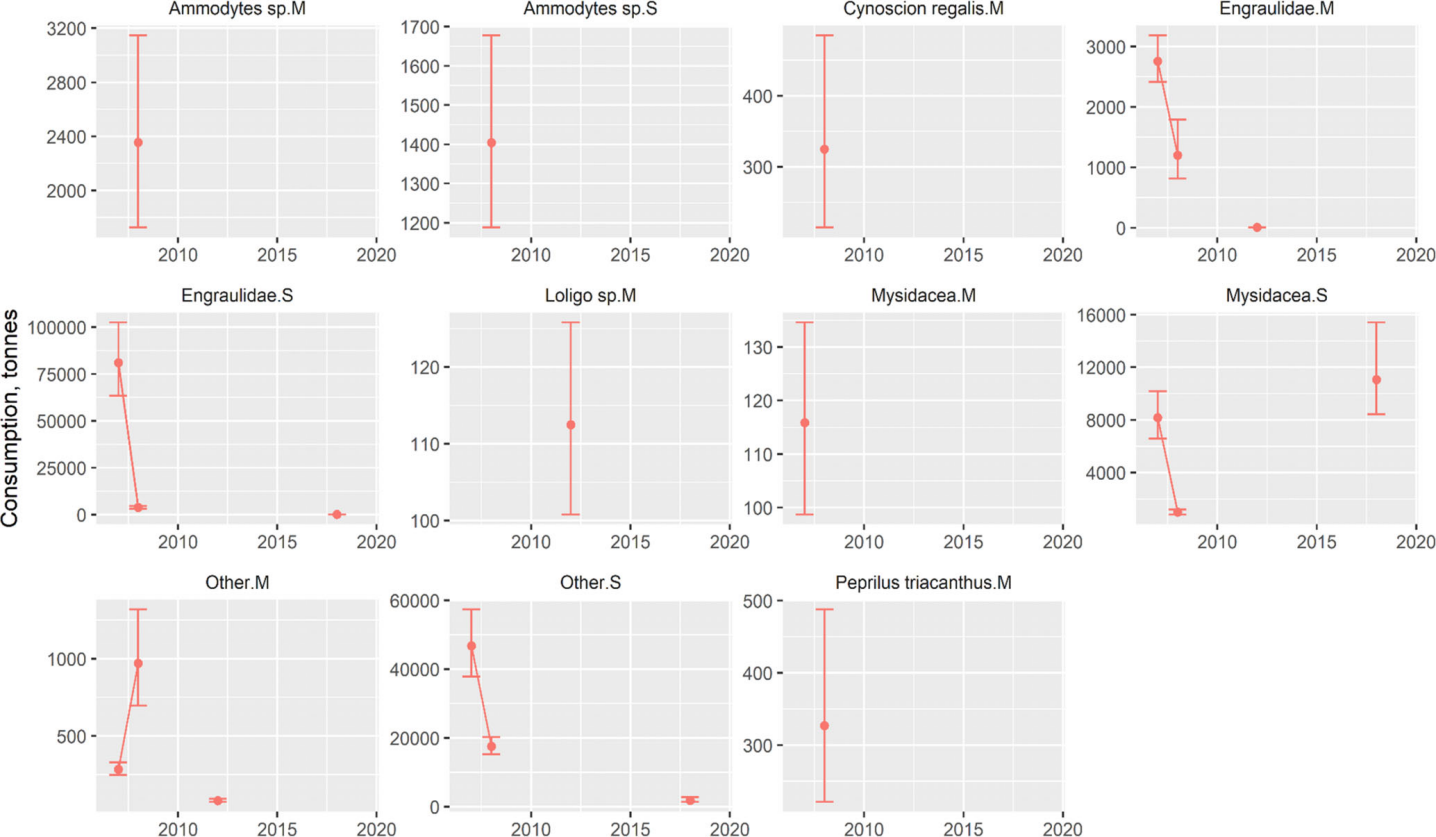
Fall time series of prey biomass removed by weakfish offshore (MAB). Error bars denote 95% confidence intervals. M, medium; S, small.

The impact of weakfish predation on dominant and commercially important prey was variable throughout 2007–2019. For each of the three areas by season and weakfish size category, the fall was the primary season when predation occurred with inshore waters having the largest amounts of prey biomass removed (Figures 6, 7, 8). In CB, time series averages of Atlantic menhaden (120.3 t per year), Engraulidae (72.5 t per year), spot (792.0 t per year), and other prey (121.3 t per year) were removed by medium weakfish in the fall, although this mostly disappeared after 2010 (Figure 6). Small weakfish in the fall were also eating Atlantic menhaden (59.7 t per year), weakfish cannibalism (30.3 t per year), Engraulidae (997.1 t per year), spot (303.5 t per year), Mysidacea (26.6 t per year), and other prey (139.8 t per year). Other prey consisting of mostly unidentified fishes and well‐digested animal remains had a greater presence with small weakfish owing to the challenges of visually identifying digested prey of smaller predators but was relatively consistent throughout the time series in CB (Figure 6). For inshore waters, again, impact on Atlantic menhaden in the fall occurred early in the time series (medium: 1020.4 t per year; small: 1409.1 t per year; Figure 7). Weakfish cannibalism and Engraulidae predation, aside from an occasional year of peak predation, remained relatively constant at 2297.7 t and 3248.3 t per year, respectively, for medium‐sized weakfish. For small weakfish, they ate 3.0 t per year of weakfish (cannibalism) and 32,208.2 t per year of Engraulidae. In the middle of the time series, medium weakfish were also eating *Loligo* spp. and other prey, and small weakfish were eating Mysidacea and other prey. Toward the end of the time series, trophic impact on silver hake (127.7 t per year) and scup (106.9 t per year) increased in magnitude for medium‐sized weakfish. The trophic impact of weakfish in the offshore waters was sporadic and limited to the fall season due to their absence in these waters during the spring (Figure 8). Minimal information, aside from a few data points for several prey, was available, emphasizing a lesser importance of this offshore monitoring programme for managing the weakfish population. However, the impact on ecologically important forage fishes by weakfish size categories (Engraulids: small 28,390.7 t per year and medium: 1320.2 t per year) and Mysidacea by small weakfish (6747.3 t per year) is worth noting for these waters.

## 4 DISCUSSION

Our study examined weakfish diet within three major geographic areas along the Northwest Atlantic and showed that in each area weakfish primarily consumed Engraulidae, Mysidacea, and bony fish, with the majority of predation occurring inshore given their population distribution. These results also show within each area, smaller weakfish have a preference for small Crustacea, whereas bigger weakfish consume larger prey (e.g., spot, Atlantic menhaden, *Loligo* spp. and *Ammodytes* spp.); however, small weakfish provide greater trophic impact to some key forage fishes (e.g., Engraulidae), particularly along the coast. Our results agree with the previous work that suggested a diet primarily composed of bony fish and crustaceans, such as those observed here (Garrison & Link, 2000; Hartman & Brandt, 1995; Parthree, 2005; Smith & Link, 2010; Willis et al., 2015; Wuenschel et al., 2013). Interestingly but agreeing with Smith and Link (2010), other commercially important species such as butterfish, silver hake, and scup were not found as much as the other identified prey. This could be due to the fact that data were limited for larger size classes of weakfish, which could be consuming these prey in higher numbers and warrant further sampling. For example, large‐sized weakfish consumed more scup, and to a lesser degree silver hake, compared to smaller individuals (Smith & Link, 2010). Large individuals, along with other piscivores that have dietary overlap with weakfish, selectively prey on Atlantic menhaden (Hartman & Brandt, 1995; Parthree, 2005; Smith & Link, 2010; Wuenschel et al., 2013). Substantial fishing of species, such as Atlantic menhaden and/or population declines, could affect the production of weakfish and other large piscivorous species. It is feasible that several other commercially important species occurred at larger proportions in the diet of weakfish, but given the challenges of identifying highly digested prey (Willis et al., 2015), these could only be identified as bony fish. In our study, Mysidacea and bony fish were very common in the diet of weakfish. This could be an indicator of prey availability and preference (Fahrig et al., 1993; Lasley‐Rasher et al., 2015; Link, 2004), which have been seen as important mechanisms driving ontogenetic diet shifts in fishes (e.g., Choi & Suk, 2012; Hjelm et al., 2000; Kimirei et al., 2013; Sánchez‐Hernández & Cobo, 2018). However, organisms such as Mysidacea that occur in high numbers due to their small size, aggregational behavior (Omori & Hamner, 1982), and diel vertical migrations (Robertson & Howard, 1978) could provide more opportunities for predatory encounters (Goldman & Sedberry, 2010) rather than prey preference or nutritional value for weakfish. Various fishes have been found to be the largest dietary component of weakfish by weight (Willis et al., 2015), and weight has a greater relevance when it comes to measuring the trophic impact of a species (as shown here) and the energetic contribution of the prey in the diets of fish (Bowen, 1983; Chipps & Garvey, 2007).

Understanding the factors and mechanisms that influence the diets of fish is important for monitoring and managing various ecosystems (e.g., Byron & Link, 2010; Latour et al., 2008; Smith & Smith, 2020). For weakfish, our results showed that season and size played a crucial role as factors that determine the variation in diet, and this was observed in the CB and inshore areas. Even though the prey items mentioned previously were the most dominant and similar throughout the mainland to offshore gradient, weakfish did show preference for spot and Atlantic menhaden toward inshore and CB. Previous studies have not examined weakfish diet across multiple spatial locations to identify patterns of change. However, a study found that prey importance varied for weakfish during the sampling time frame (2004–2005) within the James, York, and Rappahannock rivers in Virginia (Parthree, 2005). Similar findings considering sampling areas have been seen in some of weakfish's natural competitors. For example, bluefish were found to be predominantly piscivorous at reef sites than at non‐reef sites in the Chesapeake Bay (Harding & Mann, 2001). Also, the diet of striped bass has been found to also vary spatially and temporally within regions of the Chesapeake Bay (Overton et al., 2009). On the contrary, previous studies showed that size explained a substantial amount of diet variation for weakfish and other piscivores. It has been found previously that weakfish diet shifts as individuals get older and larger, with the importance of crustaceans decreasing at later ages (Hartman & Brandt, 1995; Merriner, 1975). Similarly, weakfish incorporated more fish and squid in their diet as they increased in size on the inner continental shelf off New Jersey (Wuenschel et al., 2013), and this same pattern was observed by this study and expanded to the Chesapeake Bay. Overton et al. (2009) showed that the diet of striped bass varied within size classes. Ontogenetic differences in diet and greater amounts of piscivory with increasing size (similar to weakfish) were observed for summer flounder in the Chesapeake Bay (Latour et al., 2008). Although this is not surprising because both have been found to be important independently in previous work, we found that with overlap and connectivity among the areas sampled, size had a slightly stronger influence on the diet variation in weakfish compared to season in the inshore area, whereas their influence was more equal in CB. The annual diet variability observed here was not significant, suggesting that the prey field did not significantly change during this period (2007–2019). This supports previous findings, where time over decades has not explained a significant amount of prey variation for other fish predators of this continental shelf (e.g., benthivores; Byron & Link, 2010; Rowe & Smith, 2022).

Future work would benefit from looking at temporal variability over multiple decades when additional data are available. It would also be worthwhile to examine weakfish diet variation latitudinally within each broad geographic area considered here. Although this was outside the scope of this study, and diet sampling across the three monitoring programmes was designed to capture dietary change over large spatial areas, particularly for the offshore environment, additional sources of variation considering geography are possible. At this time, the diet data were too limited to address more than the three factors considered here concurrently. Despite the differences in field sampling methods between areas (microscopic vs. macroscopic prey identification), the challenges of prey identification were omnipresent (e.g., unidentified fish prey). This caveat can be common with diet studies, particularly ones of broad scope (many predators; Smith & Link, 2010) and spatial scale (Latour et al., 2008; Wuenschel et al., 2013), and emphasizes the value of nontraditional approaches to prey identification (Paquin et al., 2014; Pitchford et al., 2020).

Weakfish prey biomass removal occurs primarily in coastal inshore habitats and notably for small‐sized weakfish considering the various prey selected. This has implications for fisheries with regard to cannibalism and the abundance of commercially important species inshore that have a crucial role as forage species (e.g., Atlantic menhaden; Anstead et al., 2021). Cannibalism in weakfish has been pointed out in previous studies (Merriner, 1975; Parthree, 2005; Wuenschel et al., 2013). Our results show that cannibalism is an essential part of the weakfish diet. It was primarily observed in coastal inshore habitats, where we estimated a fall mean biomass of cannibalism for medium weakfish at 2297.7 t per year and for small weakfish at 1285.5 t per year, with a maximum biomass of 12,517.4 t per year for medium weakfish and 4481.0 t per year for small weakfish. Considering weakfish natural mortality, this work provides a magnitude of weakfish cannibalism offering insight into quantifying natural mortality with empirical data.

The reason for weakfish shifting to cannibalism is not completely known. Possible causes of natural mortality mentioned in recent stock assessments (e.g., starvation and lack of prey) could be a driver of cannibalism. Competition with other piscivores could also trigger a cannibalistic diet. Important forage prey, such as Engraulidae, Mysidacea, and bony fish, are not necessarily federally managed or of commercial interest, but they were abundant in our findings and consistent with previous studies on the diet of other commercially important predators such as bluefish, striped bass (Hartman & Brandt, 1995), and summer flounder (Latour et al., 2008). Strong overlap in diet and habitat between the previously mentioned predators has been observed in the inner continental shelf off New Jersey (Wuenschel et al., 2013). It has also been suggested that bluefish are a more successful predator than weakfish (Hartman & Brandt, 1995). All of this evidence suggests direct competition over prey resources and could force weakfish into cannibalism. As shown here, rates of cannibalism and Engraulidae predation by medium‐sized weakfish were higher at the start of the time series in contrast to Atlantic menhaden and Mysidacea predation. Later in the time series, this pattern switched to lower rates of cannibalism and Engraulidae predation with higher rates of menhaden and Mysidacea predation. A similar prey switching occurred with small‐sized weakfish, albeit the rates of Engraulidae predation were in contrast to cannibalism, menhaden, and Mysidacea. Natural mortality on weakfish has remained high in the early 2000s, including the period that reflected higher cannibalism in our time series, particularly inshore (ASMFC, 2021). The abundance of juvenile Atlantic menhaden and recruitment decreased during this same period (ASMFC, 2022) followed by an increase in recruitment in 2010, which was reflected by the increased rates of menhaden consumption here. These changes in prey availability could lead to cannibalism. Regardless, cannibalism generally targets the juveniles of a population and can result in notable impacts, particularly with fish population dynamics (e.g., alter size distributions, age structure and growth rates, and influence recruitment; Link et al., 2012).

Some of the main factors that determine the structure and function of ecosystems are the interactions that occur between predators and prey and among predators (Braga et al., 2012; Schmitz, 2007; Tam et al., 2017). The dietary results shown here represent a comprehensive view of the common and essential prey demands of weakfish across spatial, ontogenetic, and temporal scales. Also, the opportunistic feeding nature of weakfish is highlighted in our results and is consistent with Parthree (2005) and Willis et al. (2015). Similar to other piscivores of this Northwest Atlantic region (Smith & Smith, 2020), weakfish appear to regularly switch fish prey, likely due to the relative abundance of various prey selected by weakfish. For future work to provide better estimates of prey removal, it would be worth looking more into the stomach contents of large size classes and use other nonvisual identification methods (e.g., DNA‐based; Pitchford et al., 2020) to decrease the amount of unidentified bony fish and verify if remains belong to species of commercial and/or federal interest. With the concern that weakfish is a highly piscivorous sciaenid that includes commercially important species in their diet (e.g., Atlantic menhaden, butterfish, scup, Atlantic herring, silver hake, spot, *Loligo* spp., cannibalism [Garrison & Link, 2000; Hartman & Brandt, 1995; Parthree, 2005; Smith & Link, 2010; Taylor, 1987; Willis et al., 2015; Wuenschel et al., 2013]) and have the potential to regulate energy flow in lower trophic levels, our results suggest that there is not a strong influence by weakfish on federally managed prey considered in this work. Estimates of weakfish biomass removed (cannibalism) will be informative for the weakfish stock assessment when considering natural mortality and with regard to understanding temporal patterns in weakfish recruitment. This information could also help improve ecosystem‐level models when considering competition with commercial species that share similar habitats and diets with weakfish across the Northwest Atlantic and large estuarine environments, such as Chesapeake Bay.

## AUTHOR CONTRIBUTIONS

Brian E. Smith conceived the project. Angel Reyes Delgado and Brian E. Smith made equal contributions to all aspects of data handling, analysis, and manuscript preparation.

## FUNDING INFORMATION

This study was funded by National Oceanic and Atmospheric Administration, Office of Education, Educational Partnership Program, Award numbers: NA16SEC4810007; NA21SEC4810005.

## Supporting information


**FIGURE S1** Trophic diversity curves for a number of stomachs of weakfish. Black line represents mean Shannon H. Gray shading denotes standard deviation. Labeled values indicate asymptotic diversity as the difference between the last value and average of the five preceding values. CB = Chesapeake Bay; S = small; M = medium; a = 2007/fall/M; b = 2007/fall/S; c = 2007/spring/M, d = 2007/spring/S; e = 2008/fall/M; f = 2008/fall/S; g = 2008/spring/S; h = 2009/fall/S; i = 2010/fall/M; j = 2010/fall/S; k = 2010/spring/S; l = 2011/fall/S; m = 2011/spring/S; n = 2012/fall/S; o = 2012/spring/S; p = 2013/fall/S; q = 2013/spring/S; r = 2014/fall/S; s = 2015/fall/S; t = 2015/spring/S; u = 2016/fall/M; v = 2016/fall/S; w = 2016/spring/S; x = 2017/fall/S; y = 2017/spring/S; z = 2018/fall/S; aa = 2018/spring/S.

**FIGURE S2** Trophic diversity curves for a number of stomachs of weakfish. Black line represents mean Shannon H. Gray shading denotes standard deviation. Labeled values indicate asymptotic diversity as the difference between the last value and average of the five preceding values. Coast = inshore; S = small; M = medium; a = 2007/fall/M; b = 2007/fall/S; c = 2008/fall/M; d = 2008/fall/S; e = 2008/spring/S; f = 2009/fall/M; g = 2009/fall/S; h = 2009/spring/S; i = 2010/fall/M; j = 2010/fall/S; k = 2010/spring/S; l = 2011/fall/M; m = 2011/fall/S; n = 2011/spring/S; o = 2012/fall/M; p = 2012/fall/S; q = 2012/spring/S; r = 2013/fall/M; s = 2013/fall/S; t = 2013/spring/M; u = 2013/fall/S; v = 2014/fall/M; w = 2014/fall/S; x = 2014/spring/M; y = 2014/spring/S; z = 2015/fall/M; aa = 2015/fall/S; bb = 2015/spring/S; cc = 2016/fall/M; dd = 2016/fall/S; ee = 2016/spring/M; ff = 2016/spring/S; gg = 2017/fall/M. hh = 2017/fall/S; ii = 2018/fall/M; jj = 2018/fall/S; kk = 2018/spring/S; ll = 2019/fall/M; mm = 2019/fall/S; nn = 2019/spring/M; oo = 2019/spring/S.

**FIGURE S3** Trophic diversity curves for a number of stomachs of weakfish. Black line represents mean Shannon H. Gray shading denotes standard deviation. Labeled values indicate asymptotic diversity as the difference between the last value and average of the five preceding values. MAB = Mid‐Atlantic Bight/offshore; S = small. M = medium. a = 2007/fall/M; b = 2007/fall/S; c = 2008/fall/M; d = 2008/fall/S; e = 2012/fall/M; f = 2018/fall/S.
