# Supplementary figures and images for: Diet variation and trophic impact of weakfish, Cynoscion regalis, within multiple marine habitats of the eastern United States
Source: J Fish Biol. 2024 Aug 12;105(6):1628–42. doi: 10.1111/jfb.15897 (PMC11650933; doi:10.1111/jfb.15897)

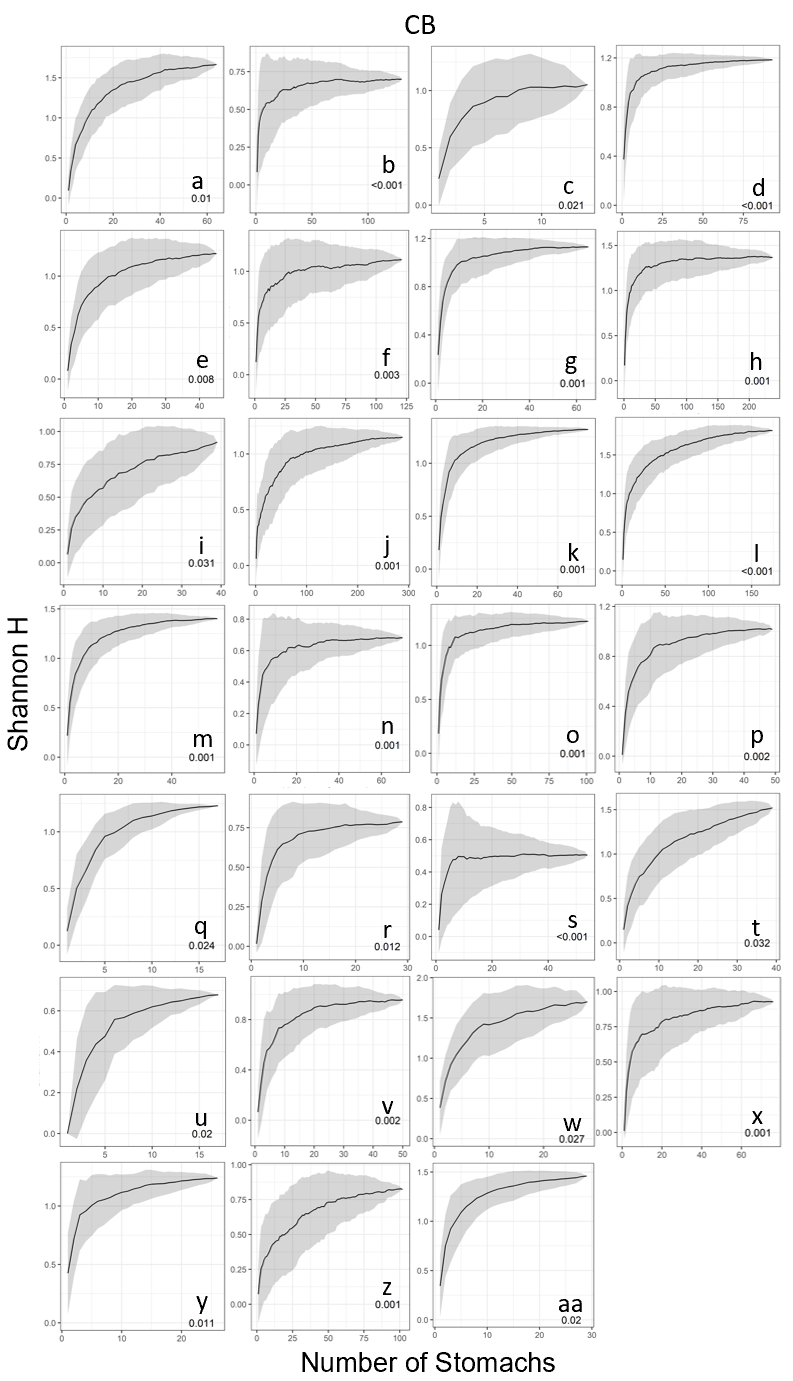

Supplement: Supplementary file 1 — FIGURE S1 Trophic diversity curves for a number of stomachs of weakfish. Black line represents mean Shannon H. Gray shading denotes standard deviation. Labeled values indicate asymptotic diversity as the difference between the last value and average of the five preceding values. CB = Chesapeake Bay; S = small; M = medium; a = 2007/fall/M; b = 2007/fall/S; c = 2007/spring/M, d = 2007/spring/S; e = 2008/fall/M; f = 2008/fall/S; g = 2008/spring/S; h = 2009/fall/S; i = 2010/fall/M; j = 2010/fall/S; k = 2010/spring/S; l = 2011/fall/S; m = 2011/spring/S; n = 2012/fall/S; o = 2012/spring/S; p = 2013/fall/S; q = 2013/spring/S; r = 2014/fall/S; s = 2015/fall/S; t = 2015/spring/S; u = 2016/fall/M; v = 2016/fall/S; w = 2016/spring/S; x = 2017/fall/S; y = 2017/spring/S; z = 2018/fall/S; aa = 2018/spring/S. [file JFB-105-1628-s002.docx]

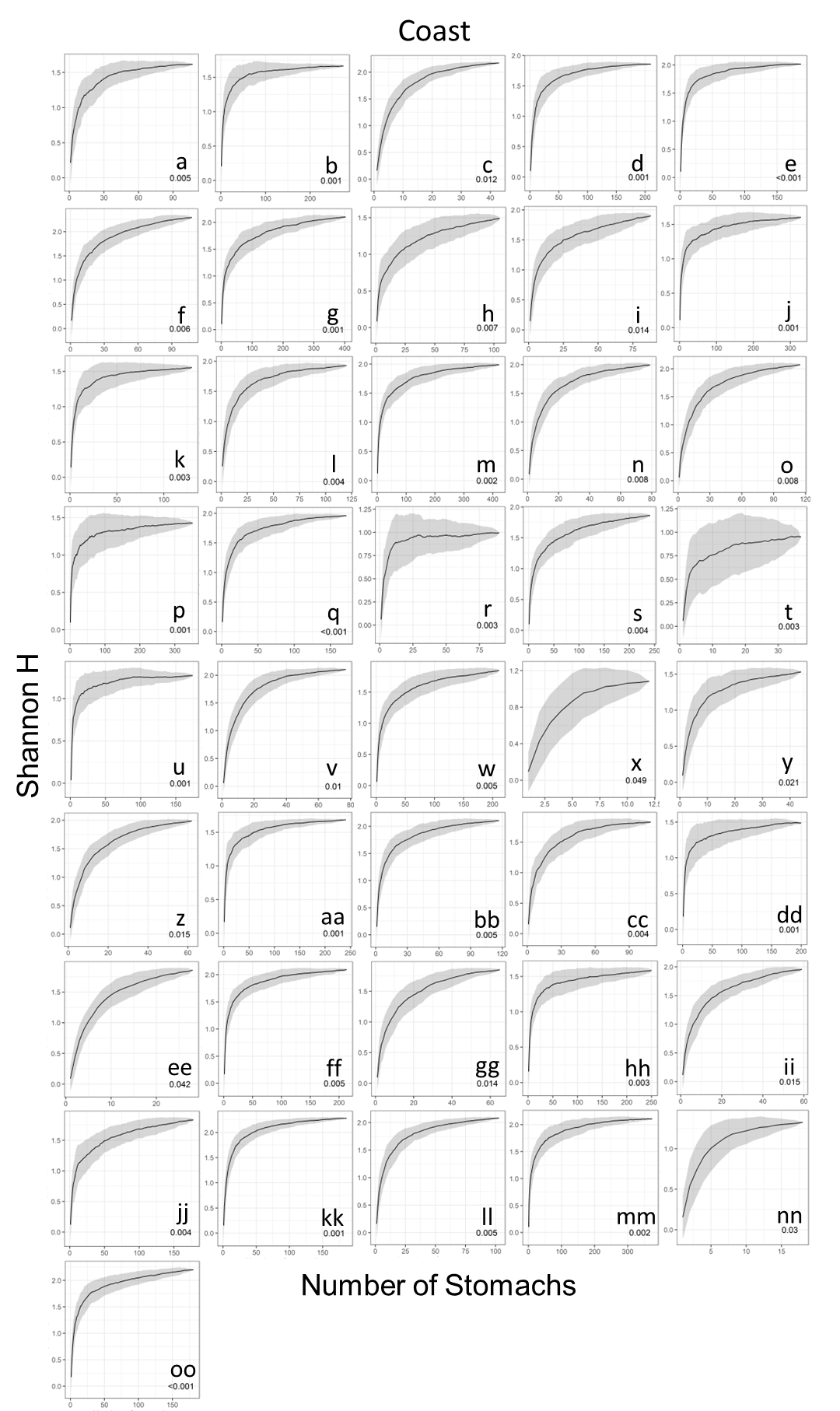

Supplement: Supplementary file 2 — FIGURE S2 Trophic diversity curves for a number of stomachs of weakfish. Black line represents mean Shannon H. Gray shading denotes standard deviation. Labeled values indicate asymptotic diversity as the difference between the last value and average of the five preceding values. Coast = inshore; S = small; M = medium; a = 2007/fall/M; b = 2007/fall/S; c = 2008/fall/M; d = 2008/fall/S; e = 2008/spring/S; f = 2009/fall/M; g = 2009/fall/S; h = 2009/spring/S; i = 2010/fall/M; j = 2010/fall/S; k = 2010/spring/S; l = 2011/fall/M; m = 2011/fall/S; n = 2011/spring/S; o = 2012/fall/M; p = 2012/fall/S; q = 2012/spring/S; r = 2013/fall/M; s = 2013/fall/S; t = 2013/spring/M; u = 2013/fall/S; v = 2014/fall/M; w = 2014/fall/S; x = 2014/spring/M; y = 2014/spring/S; z = 2015/fall/M; aa = 2015/fall/S; bb = 2015/spring/S; cc = 2016/fall/M; dd = 2016/fall/S; ee = 2016/spring/M; ff = 2016/spring/S; gg = 2017/fall/M. hh = 2017/fall/S; ii = 2018/fall/M; jj = 2018/fall/S; kk = 2018/spring/S; ll = 2019/fall/M; mm = 2019/fall/S; nn = 2019/spring/M; oo = 2019/spring/S. [file JFB-105-1628-s001.docx]

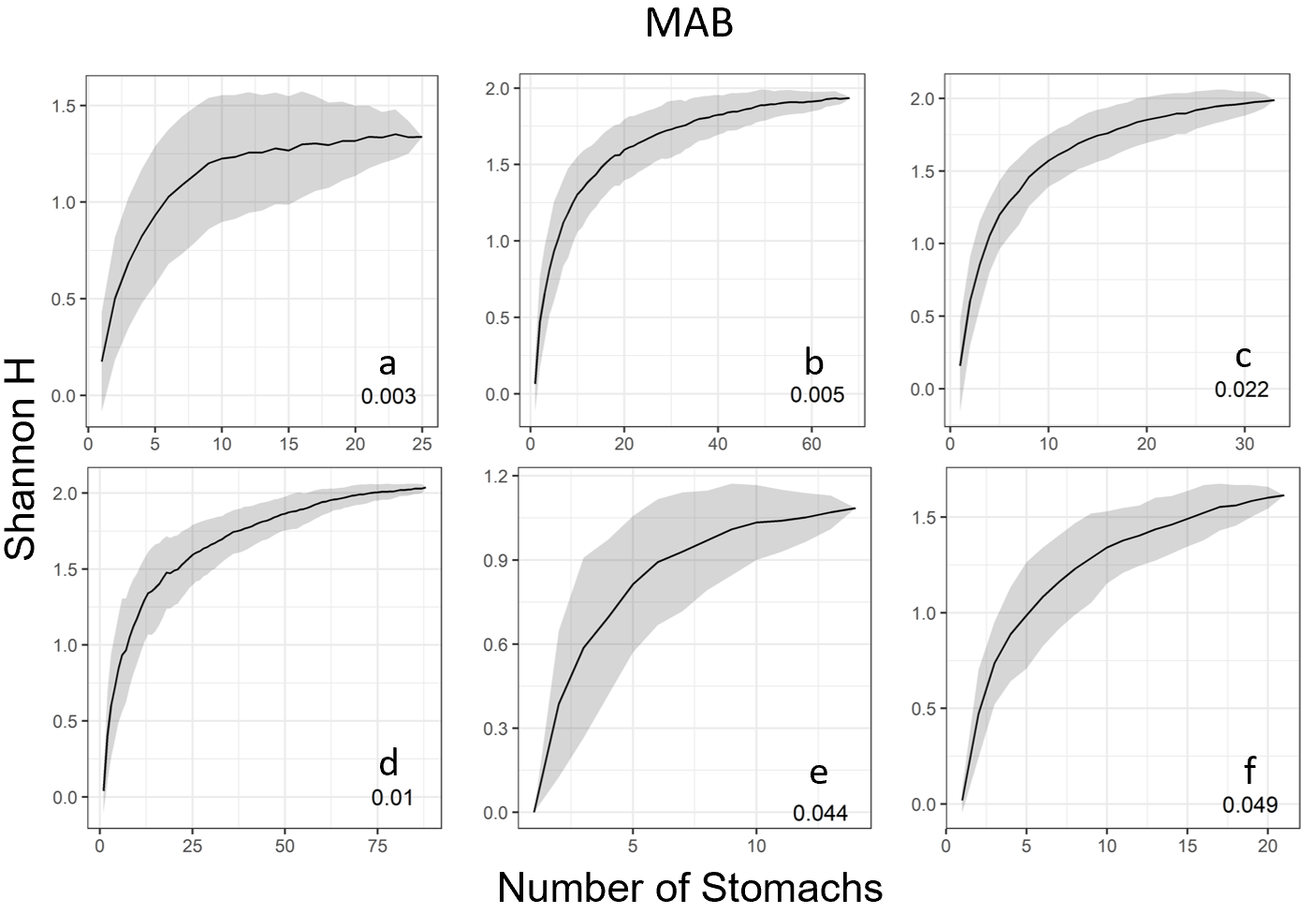

Supplement: Supplementary file 3 — FIGURE S3 Trophic diversity curves for a number of stomachs of weakfish. Black line represents mean Shannon H. Gray shading denotes standard deviation. Labeled values indicate asymptotic diversity as the difference between the last value and average of the five preceding values. MAB = Mid‐Atlantic Bight/offshore; S = small. M = medium. a = 2007/fall/M; b = 2007/fall/S; c = 2008/fall/M; d = 2008/fall/S; e = 2012/fall/M; f = 2018/fall/S. [file JFB-105-1628-s003.docx]
